# Environment-Driven Adaptations of Leaf Cuticular Waxes Are Inheritable for *Medicago ruthenica*

**DOI:** 10.3389/fpls.2021.620245

**Published:** 2021-05-17

**Authors:** Yanjun Guo, Xiao Zhao, Yang Li, Zhen Li, Qianlin Xiao, Yanmei Wang, Xuefeng Zhang, Yu Ni

**Affiliations:** ^1^College of Animal Science and Technology, Southwest University, Chongqing, China; ^2^College of Agronomy and Biotechnology, Southwest University, Chongqing, China

**Keywords:** adaptation, climate change, cuticular wax, epigenetic modifications, genetic variations, *Medicago ruthenica*

## Abstract

Cuticular waxes covering the plant surface play pivotal roles in helping plants adapt to changing environments. However, it is still not clear whether the responses of plant cuticular waxes to their growing environments are inheritable. We collected seeds of *Medicago ruthenica* (a perennial legume) populations from 30 growing sites in northern China and examined the variations of leaf cuticular waxes in a common garden experiment. Four wax genes, *MrFAR3-1, MrFAR3-2*, *MrCER1*, and *MrKCS1*, involved in biosynthesis of predominant wax classes (primary alcohol and alkane) and wax precursors, were isolated to test the contributions of genetic variations of the coding sequences (CDS) and the promoter sequences and epigenetic modifications. The plasticity responses of the cuticular waxes were further validated by two stress-modeling experiments (drought and enhancing ultraviolet B). Great variations in total wax coverage and abundance of wax classes or wax compounds were observed among *M. ruthenica* populations in a common garden experiment. Stress-modeling experiments further validated that *M. ruthenica* would alter leaf wax depositions under changed growing conditions. The transcriptional levels of the wax genes were positively or negatively correlated with amounts of cuticular waxes. However, the analysis of promoter methylation showed that the methylation level of the promoter region was not associated with their expressions. Although both promoter sequences and CDS showed a number of polymorphic sites, the promoters were not naturally selected and insignificant difference could be observed in the numbers and types of acting elements of the four wax genes among populations. In contrast, the CDS of the wax genes were naturally selected, with a number of missense mutations resulting in alterations of the amino acid as well as their isoelectric points and polarities, which could impact on enzyme function/activity. We conclude that long-term adaptation under certain environments would induce genetic mutation of wax biosynthesis genes, resulting in inheritable alterations of cuticular wax depositions.

## Introduction

Cuticular waxes interact with the environments and protect plants from abiotic and biotic stresses (Wertz, 1996). These hydrophobic compounds cover the epidemic cells forming wax crystals or films and are embedded within cutin matrix (Jeffree, 2006). For the vast vascular plants, the formation of cuticle has been shown to be one of the most successful evolutionary products, which prevents evaporation of water from the epidermal surface and external water and solutes from entering the tissues, enabling them to survive through changing environments (Yeats and Rose, 2013). Both stress-modeling experiments and natural environmental investigations have shown that plant cuticular waxes are sensitive to changing environments (Dodd and Poveda, 2003; Shepherd and Griffiths, 2006; Kosma et al., 2009). However, the mechanisms that contribute to the wax variations between plant populations located at different environments are still not clear.

Cuticular waxes consist of long-chain fatty acids and their derivatives such as aldehydes, primary alcohols, alkyl esters, alkanes, secondary alcohols, ketones, triterpenoids, and diketones, etc. (Jetter et al., 2006). Under stressed conditions, plants will adjust both the quantity and the relative abundance of wax compositions to adapt to changing environments. For example, drought will increase the total wax load of the most tested plant species, and an increase of alkane content has been shown to be a common response of plant wax to drought stress (Shepherd et al., 1997; Kim et al., 2007). Enhanced ultraviolet B (UVB) increased the proportion of nonacosane diols on Norway spruce needles but reduced that of alkyl esters (Gordon et al., 1998). The epicuticular wax profiles of *Dianthus caryophyllus* from a greenhouse showed a distinct shift in wax compounds with developmental age and type of box that was used (vented or not; Correll and Weathers, 2001). However, up to now, no direct evidence has proved how much wax deposition is required for certain plant species under certain environments. Plants growing under the same environment differ greatly in their wax amounts and wax compositions (Gao et al., 2016; He et al., 2016), implying that plant species with low wax deposition and high wax deposition can adapt to similar environments. Unlike enzyme activities and osmoprotectants that are reversible in plants, the environment-induced wax deposition is irreversible. This is helpful for plants that confront similar frequent stresses during their growing stages. A study on populations of *Juniperus communis* var. saxitilis and var. montana from the Pyrenees, the Alps, Corsica, and California has shown that plants adjust chain length distribution of alkanes to adapt to their growing conditions (Dodd and Poveda, 2003). A study on populations of *Plantago major* has also shown that the populations of *P. major* can be separated according to the average annual temperature of their habitats based on cluster analysis using the pattern of the *n*-alkane distributions (Guo et al., 2015). To investigate the possible correlations between habitat and wax variations, Dodd and Afzal-Rafii (2000) analyzed three species from the family Cupressaceae (*Austrocedrus chilensis*, *Fitzroya cupressoides*, and *Pilgerodendron uviferm*) in different climatic conditions and concluded that natural selection had favored cuticular hydrocarbon mixes that provided differential fitness in the face of habitat differences in water stress and temperature. However, whether such variations of cuticular wax under various climatic conditions are heritable is still unknown.

Recently, a study from a common garden experiment has shown that wax deposition patterns of 59 populations of *Leymus chinensis* formed during their adaptations to long-term growing environments could not inherit in their progenies and exhibit such inheritance even if these progenies were exported to new environments (Li et al., 2020). Similar results were also obtained from studies with *Melaleuca quinquenervia* across eastern Australia, where *n*-alkane characteristics could be driven by genetic differences between populations (Andrae et al., 2019) and from *Eucalyptus globulus* where the variation and differentiation in cuticular wax compounds had a complex genetic origin (Gosney et al., 2016). However, molecular mechanisms behind amount and composition variations of cuticular waxes are needed to clarify the genetic origins among plant populations distributed under various environments.

The biosynthesis of cuticular waxes occurs in endoplasmic reticulum (ER), where C_16_ and C_18_ fatty acyl precursors produced in epidermal plastids are activated to acyl-CoAs (Samuels et al., 2008). Then, the chain length of these acyl-CoAs are elongated by fatty acid elongase complexes consisted of a ketoacyl-CoA synthase (KCS), a ketoacyl-CoA reductase, a hydroxyacyl-CoA dehydratase, and an enoyl-CoA reductase. The long-chain acyl-CoAs are further catalyzed to alcohols that are esterified with fatty acids through head group reduction (alcohol forming pathway) and to aldehydes, alkanes, secondary alcohols, and ketones through head group reduction, decarbonylation, and oxidation (alkane forming pathway; Samuels et al., 2008). In some plant species, type III polyketide synthases are involved in synthesizing β-diketones (Kosma and Rowland, 2016). The identification of genes involved in wax biosynthesis in *Arabidopsis thaliana* and in other crops greatly improves our understanding of the functions of cuticular wax. However, it is still a challenge for plants lacking genome data to investigate their wax genes, particularly those mainly distributed in natural environments.

**FIGURE 1:**
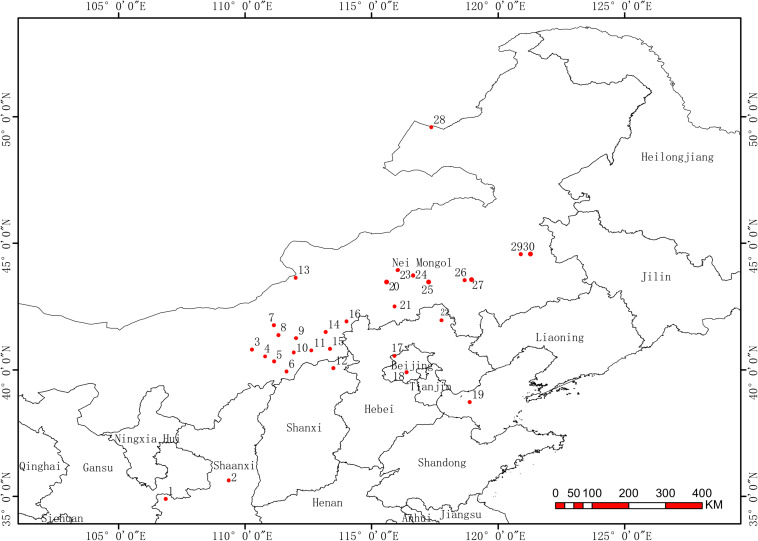
The original locations of 30 *Medicago ruthenica* populations.

*Medicago ruthenica* (2n), a perennial legume forage from genus Medicago, is widely distributed in steppes with annual precipitation ranging from 250 to 400 mm. Studies have shown that this plant has wide adaptations to various environments and exhibits high morphological and genetic diversities among populations (Li et al., 2016; Hao et al., 2017). In our pre-experiment, we observed that the cuticular waxes on leaves of *M. ruthenica* mainly consisted of primary alcohol and alkanes. In order to explore whether the variations of cuticular wax under various climatic conditions are heritable, we collected seeds of *M. ruthenica* from 30 growing sites with longitudes ranging from E106 to E120 and latitudes ranging from N34 to N49 (Figure 1). We isolated four wax genes (*MrFAR3-1, MrFAR3-2, MrCER1*, and *MrKCS1*) and their promoters from *M. ruthenica*, which are responsible for the biosynthesis of primary alcohol and alkane (predominant wax classes) and wax precursors. Then, in a common garden experiment, we further analyzed the leaf morphological parameters (length, width, length/width, area, and angle of leaflets), variations of leaf cuticular waxes, and transcriptional level and promoter methylation level of the four wax genes in 30 populations. In order to investigate the genetic variations of these isolated genes, the diversity of the coding sequences (CDS) and the promoter sequences of the four wax genes was further analyzed. Meanwhile, two stress modeling experiments, drought and enhancing UVB, were also applied on four populations with significant difference in wax deposition in the common garden experiment, aiming to investigate the plant performance under different environmental pressures. We hypothesized that the climate-induced variations of leaf cuticular wax among plant populations might be driven by both environment and genetic variations.

## Materials and Methods

### Experimental Materials

The seeds of *M. ruthenica* were collected from their natural growing sites (Figure 1 and Supplementary Table 1), preserved under the conditions of 4°C and 55% relative humidity in Seed Resource Bank of Grassland Research Institute of Chinese Academy of Agricultural Sciences and Ministry of Agriculture. In total, seeds from 29 populations and one released cultivar (TMT) were obtained. We investigated the seed morphological parameters including seed length, seed width, length/width ratio, and 1,000 grain weight, and found that the coefficient of variance (CV) of these parameters ranged from 5.23 to 10.91% (Supplementary Table 2). Correlation analysis of the original climatic factors with seed parameters indicated that only seed length/width was negatively correlated with annual average temperature (Supplementary Table 3).

### Common Garden Pot Experiment

Seeds of *M. ruthenica* were sterilized with 10% H_2_O_2_ for 10 min, rinsed three times under tap water, and then planted in pots (10 cm × 15 cm) filled with 2 kg of soils (vermiculite: soil = 1:2). The soil pH was 6.79 (water : soil = 5:1). To keep sufficient soil nutrients for plant growth, 0.02 g/kg nitrogen (Urea, N 46.3%), 0.10 g/kg of P_2_O_5_ (Superphosphate, P_2_O_5_ 12%), and 0.10 g/kg of K_2_O (K_2_SO_4_, K 52%), were supplemented. The pots were placed in a growth chamber (light intensity 10,000 Lx, light/dark 14 h/10 h, temperature 25°C/20°C, and humidity is 80%). The positions of the pots were adjusted every other day. In total, four plants were kept in each pot and each population had four pots. The plants were watered every 3 days to keep the soil water field capacity (FC) at 75%. 2 months after planting, when the plants were in their branching stages (height, 30–40 cm), the leaves were sampled for wax extractions, DNA and RNA extractions, and morphological measurements.

### Environmental Stress Modeling

From the common garden experiments, two populations with relatively high wax amounts (02923 and 02929) and two populations with low wax amounts (TMT and GS4193) were further selected and were subjected to drought and enhanced UV-B treatments. Seeds were sterilized with 10% H_2_O_2_ for 10 min, rinsed three times under tap water, and then planted in pots (10 cm × 15 cm) filled with 2-kg soils (vermiculite: soil = 1:2). The soil was same as the soil used in common garden experiment. Four plants were kept in each pot. The pots were placed in a greenhouse (light intensity 3,500 Lx, light/dark 14 h/10 h, temperature 25°C/20°C, and humidity is 75%) for 6 weeks and then were subjected to drought and UVB treatments. Each treatment had four replicates (pots).

For drought experiment, the plants were subjected to two soil water conditions, 75% FC (well-watered condition), and 45% FC (drought-stressed condition), for 20 days. The water was supplemented in the morning and the pot positions were adjusted every day.

For UVB experiment, the plants were subjected to two UVB conditions, 0 (control) and 0.15 W/m^2^ (enhanced UVB). The enhanced UVB was obtained by exposing the plants under fluorescent tube (model UV-B 30W; Philips, Eindhoven, Beijing, China) for 1 h each day from 13:00 PM to 14:00 PM. UV-tube was filtered with one layer of 0.13-mm cellulose diacetate film (JCS Industries Inc., LaMirada, CA, United States) to filter radiation below 280 nm. The film was changed every 3 days. The flux density of UVB at 300 nm was measured by a UV-340A UV Light Meter (Lutron, Taiwan). The dose of 0.15 W/m^2^ UVB radiation was obtained by changing the distance between the plant top and the fluorescent tube every 2 days, aiming to keep a certain distance (ca. 20 cm) between the plant top and the tube.

### Determination of Morphological Parameters

The seed length, seed width, leaf length, and leaf width were measured by vernier caliper; the weight of 1,000 seeds was measured by one thousandth electronic balance. The leaf area and leaf base angle were measured by ImageJ software (Abramoff et al., 2004). The ratio of seed length to width and the ratio of leaf length to width were calculated.

### Cuticular Wax Extraction and Compound Identification

Healthy leaves from the same leaf position (third from the top) were sampled from the common garden experiment and environmental stress-modeling experiments, respectively. Before wax extraction, photos of leaves were taken and subjected to pixel counting using the ImageJ software to determine surface areas (Abramoff et al., 2004). Then, leaves were extracted twice in 3 ml of chloroform containing 2 μg of tetracosane as internal standard at room temperature for 30 s. The extracts were dried in a nitrogen stream at 40°C, and derivatized using 20 μl of BSTFA [N,O-Bis (trimethylsilyl) Trifluoroamide] and 20 μl of pyridine (Aldrich) for 45 min at 70°C. The surplus solutions were evaporated under nitrogen and the sample was re-dissolved in 0.5 ml of chloroform for GC and GC/MS analysis.

For compound identification, the GC analysis was carried out with 9790II gas chromatograph (Fu-Li, China). The GC column was a DM-5 capillary column (30 m × 0.32 mm × 0.25 μm). Nitrogen gas served as the carrier gas. The GC oven was held at 80°C for 10 min and heated at 5°C/min to 260°C, where the temperature remained 10 min. The temperature was then heated at 2°C/min to 290°C, and further heated at 5°C/min to 320°C, where the temperature was held for 10 min. Compounds were detected with a GCMS-QP2010 Ultra Mass Spectrometric Detector (Shimadzu Corp., Kyoto, Japan) using an HP-5 MS capillary column (30 m × 0.32 mm × 0.25 μm) and He as the carrier gas. Compounds were identified by comparing their mass spectra with published data and commercialized standards including n-tetracosane, n-pentacosane, n-hexacosane, n-heptacosane, n-octacosane, n-nonacosane, n-triacontane, n-hentriacontane, n-dotriacontane, n-tritiacontane, 1-Hexacosanol, 1-Octacosanol, and 1-triacontanol (Sigma-Aldrich). Quantification was based on FID peak areas. The amount of wax was expressed as μg/cm^2^.

### DNA and RNA Isolation and cDNA Synthesis

The genomic DNA of 30 populations was extracted from fresh leaves of *M. ruthenica* at branching stage using the cetyltrimethyl ammonium bromide method (Porebski et al., 1997). Total RNA was extracted using TransZol kit (Invitrogen, China). RNA quality and quantity were assessedesize the cDN by absorption at 260 nm/280 nm and gel electrophoresis. Total RNA was reverse transcribed to synthA using Takara’s PrimeScript^TM^ RT reagent Kit with gDNA Eraser (Perfect Real Time).

### Isolation of Cuticular Wax Genes From *M. ruthenica*

Up to now, there are no genomic data and no gene involved in cuticular wax biosynthesis has been reported for *M. ruthenica*. Based on chemical compositions of cuticular wax of *M. ruthenica*, four genes mainly involved in primary alcohol and alkane biosynthesis and fatty acid elongation, including *Arabidopsis thaliana* and *M. truncatula* ortholog CER4/FAR3, CER1, and KCS1, were selected to isolate from *M. ruthenica*. First, the sequences of these *Arabidopsis* and *M. truncatula* ortholog were blasted with the *M. ruthenica* transcriptome data deposited in the NCBI under SRA accession number SRR2177371, and then the specific primers were designed and combined with RACE (rapid amplification of cDNA ends) kit primers to amplify 5’ and 3’cDNA ends of the target genes. Based on the sequencing results of the cDNA ends, the forward and reverse primers were designed to amplify the full-length cDNA sequence of the target genes. The 25-μl PCR reaction contained 22 μl of Gold-mix including super fidelity DNA polymerase (TsingKe), 2 μl of total first-strand cDNA, and 1 μl of forward and reverse primers. The PCR procedure was as follows: initial denaturation at 98°C for 3 min followed by 30 cycles, denaturation at 98°C for 10 s, annealing temperature for 10 s at 58°C, extension at 72°C for 30 s, and final extension for 5 min at 72°C in a DNA thermal cycler (BIO OVEN III). Amplified products were determined by gel electrophoresis and cloned into pMD19-T vector (Takara, Beijing, China) for sequencing (TsingKe Biological Technology Co, Ltd). The isolated genes from *M. ruthenica* were named *MrFAR3-1*, *MrFAR3-2*, *MrCER1*, and *MrKCS1*, respectively. The NCBI GenBank accession number of the four genes were BankIt2355506 Seq2 (*MrFAR3-1*), BankIt2355508 Seq3 (*MrFAR3-2*), BankIt2355487 Seq1 (*MrCER1*), and BankIt2355509 Seq4 (*MrKCS1*). The primers used in this study are listed in Supplementary Table 4.

### Chromosome Walking for Promoter Sequences Cloning

According to the cDNA sequences of *MrFAR3-1, MrFAR3-2, MrCER1*, and *MrKCS1*, promoter sequences were cloned by the chromosome walking method using gDNA from TMT as template and using two specific primers (SP1 and SP2, Supplementary Table 4). Next, using gDNA as template, the promoter sequences of the four genes were amplified from all 30 populations. PCR was carried out in 50-μl reactions containing 19 μl of PCR-Grade Water, 25 μl of 2 × Ex taq Buffer, 1.0 μl of dNTP Mix, 1.0 μl of Ex taq, 1.5 μl of forward and reverse primers (Supplementary Table 4), and 1 μl of gDNA template. PCR products were cloned into pMD19-T vector (Takara) for sequencing.

### Quantitative Real-Time PCR

The Quantitative Real-Time PCR (qRT-PCR) was performed on a Bio-Rad CFX96 Real-Time PCR Detection System using the SYBR Premix Ex Taq II (Takara, Beijing, China). The qRT-PCR was carried out in 10-μl reactions containing 5 μl of SYBR Premix Ex Taq TMII (2×), 3.4 μl of RNase-Free H_2_O, 0.3 μl of each forward and reverse primer, and 1 μl of cDNA template. The qRT-PCR was programmed in an initial step at 95°C for 30 s, followed by 39 cycles including denaturation at 95°C for 10 s, annealing at 59°C for 30 s, and extension at 65°C for 5 s. The reference gene *Actin* was used to normalize the total RNA amount. The relative expression was calculated using the 2^–ΔΔCt^ method (Wong and Medrano, 2005). All experiments were repeated in three biological and three technical replicates. Primers used for qRT-PCR are listed in Supplementary Table 4.

### Data Analysis

One-way ANOVA analysis was applied to evaluate the influence of drought and enhanced UVB stresses on amounts of cuticular wax using SPSS 13.0 (SPSS Inc., Chicago, United States). *T* test was applied to compare the difference of the relative expression of genes between stressed and control plants (SPSS 13.0).

The homology and similarity of promoter sequences and CDS of wax genes from 30 populations were analyzed by Clustalw1.83 and DNAMAN software. Then, using DNA Sequence Polymorphism (DNAsp) software version 5.0 (Rozas et al., 2017), we tested the single nucleotide diversity index, haplotype number, haplotype diversity index, and DNA polymorphism sites of promoter sequence and CDS, aiming to evaluate the genetic polymorphisms among different populations. In order to test whether these promoters and CDS followed the neutral evolutionary model, Tajima test and Fu and Li’s test were performed by DNAsp (Tajima, 1989). The Tajima test calculates and standardizes the number of segregating sites per sequence in the alignment of the target sequence and the value of nucleotide diversity of each sequence. When the test values deviated from 0 (the theoretical value), the allele frequency did not follow the neutral evolutionary model. Negative Tajima’s *D* values and Fu and Li’s *D* and *F* values were observed in the current test, suggesting that lots of low-frequency alleles existed in the tested genes, which could be used to infer population size enlargement and directional selection.

Next, the type and quantity of the key regulatory and acting elements of promoter regions of the wax genes were analyzed and predicted by the promoter database Plantcare^1^. Using MethPrimer^2^ (Li and Dahiya, 2002), the CpG Islands of the promoter region were predicted, with a criteria of CG% > 50%, observed value/expected value > 0.6, and length of CpG segments >200 bp (Gardiner-Garden and Frommer, 1987).

Finally, the mutation characteristics of the CDS were analyzed using SnapGene V5.0.5^3^. The single-nucleotide polymorphisms (SNPs) were translated to amino acids using Sequence Manipulation Suite (Stothard, 2000). Then, the amino acids before and after mutation were compared. Amino acids from missense mutations were further analyzed for their isoelectric point, polarity, and hydropathy index.

## Results

### Variations of Leaf Cuticular Waxes Among Populations From Common Garden Pot Experiment

**FIGURE 2:**
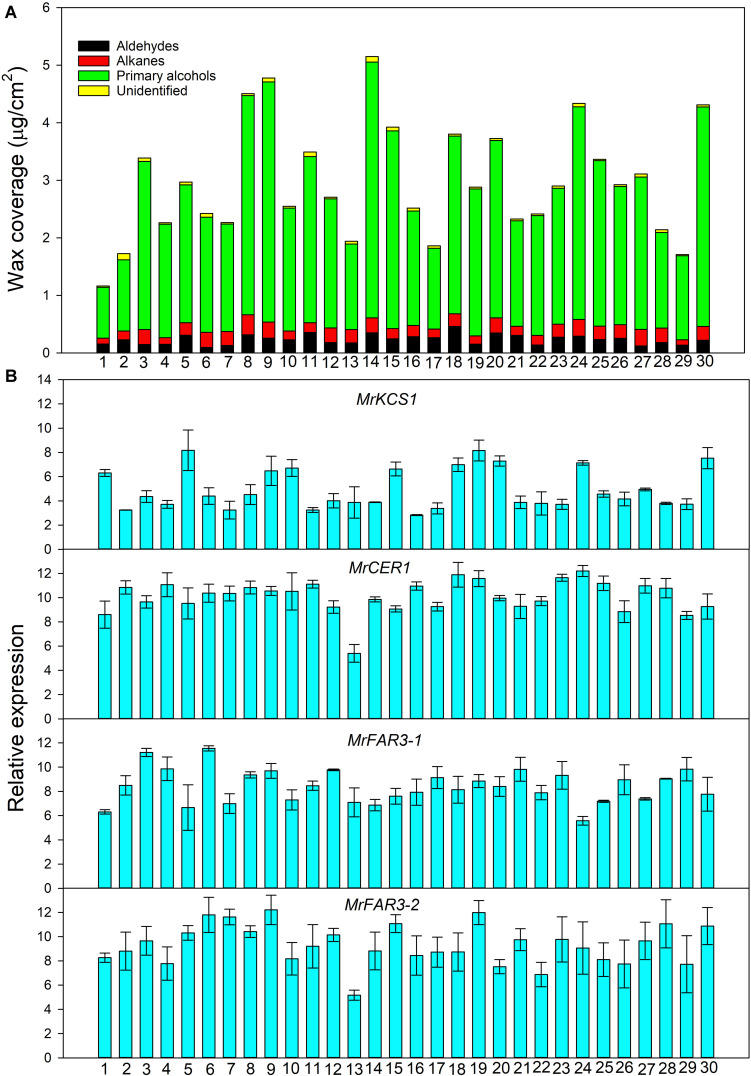
Wax coverage **(A)** and expression of genes involved in wax biosynthesis **(B)** from 30 *Medicago ruthenica* populations.

In total, three wax classes were identified in *M. ruthenica*, including aldehydes, primary alcohols, and alkanes, with the remaining unidentified (Figure 2A). The primary alcohols were the predominant compounds, with their relative abundance ranging from 69.95 to 90.56%. Great variations were observed in total wax coverage and wax classes among 30 populations (Table 1). For example, the maximum total wax coverage (6.11 μg/cm^2^) was almost six times of that of the minimum total wax coverage (1.06 μg/cm^2^). The CV reached 36.57% for total wax coverage, 32.68, 40.21, and 41.83% for alkanes, primary alcohols, and aldehydes, respectively.

**TABLE 1:** Variations of the amounts and relative abundance of wax compositions across 30 *Medicago ruthenica* populations.

Composition	Amount (μg/cm^2^)				Relative abundance (%)			
	Min.	Max.	Ave.	CV	Min.	Max.	Ave.	CV
Alkanes	0.07	0.41	0.21	32.68	3.82	16.85	7.56	33.97
Aldehydes	0.07	0.61	0.23	41.83	2.81	16.06	8.32	40.18
Alcohols	0.82	5.37	2.49	40.21	69.95	90.56	82.46	5.89
Unidentified	0.02	0.12	0.05	50.50	0.57	6.66	1.66	62.74
Total wax	1.06	6.11	2.99	36.57				
an25	0.01	0.03	0.01	33.45	2.14	15.44	6.81	42.19
an27	0.01	0.14	0.03	50.29	8.08	28.07	15.30	28.31
an29	0.02	0.24	0.07	58.03	14.26	56.56	30.43	28.73
an31	0.02	0.18	0.07	38.36	21.21	49.37	35.88	15.95
an33	0.01	0.05	0.02	36.48	7.07	16.46	11.59	17.18
26al	0.00	0.03	0.01	49.52	1.35	21.12	4.48	67.94
28al	0.06	0.56	0.21	42.65	71.75	96.62	88.90	4.43
30al	0.00	0.08	0.02	82.80	0.83	26.90	6.88	62.10
26ol	0.00	0.03	0.00	97.04	0.04	0.75	0.20	71.41
28ol	0.05	0.47	0.13	50.84	2.13	20.02	5.27	52.52
30ol	0.75	5.22	2.34	41.30	80.98	97.14	94.05	2.17
32ol	0.01	0.08	0.01	64.84	0.19	1.27	0.60	45.43

Each wax class consisted of serials of homologs with changing chain lengths. For example, the chain length of aldehydes ranged from C_26_ to C_30_, with C_28_ being the predominant compound (relative abundance, 88.9%) for all populations. Only odd carbon number alkanes were observed, with chain length ranging from C_25_ to C_33_ and C_31_ being the predominant compound (35.88%). The primary alcohols consisted of only even carbon numbers, with chain length ranging from C_26_ to C_32_ and C_30_ being the predominant compound (94.05%). Though the chain length distributions of these wax classes were similar among populations, their relative abundance differed greatly in CV%. The relative abundance of C_28_ aldehyde and C_30_ primary alcohol had the lowest CV% (4.43 and 2.17%), whereas C_26_ primary alcohol and C_26_ aldehyde had the highest CV% (71.41 and 67.94%). The CV% of alkanes ranged from 15.79% (C_33_) to 42.19% (C_25_; Table 1).

Pearson’s correlation analysis indicated that no significant relationship could be observed between the wax amounts and the environmental factors such as longitude, latitude, annual average temperature, precipitation, or arid index (data not shown).

### Variations of Gene Transcriptions and Their Relationship With Cuticular Waxes

Quantitative Real-Time PCR analysis showed that the expression level of the wax genes varied greatly among 30 populations (Figure 2B). For example, the highest relative expression level reached 8.17 for *MrKCS1* (involved in wax precursor biosynthesis), 11.89 for *MrCER1* (involved in alkane biosynthesis), 11.55 for *MrFAR3-1*, and 12.21 for *MrFAR3-2* (involved in primary alcohol biosynthesis), whereas the lowest relative expression levels of these genes were 2.82, 5.40, 5.57, and 5.17, respectively.

**FIGURE 3:**
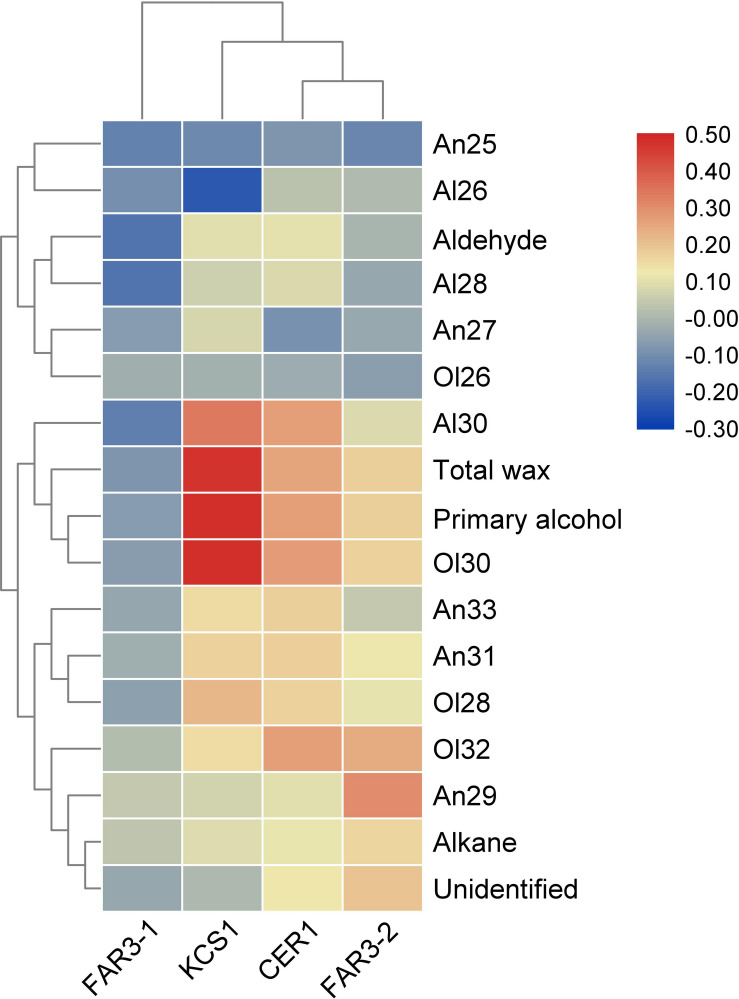
Heatmap of the Pearson’s correlation between cuticular wax and the expression of four genes involved in cuticular wax biosynthesis.

Pearson’s correlation analysis indicated that the relative expression levels of these wax genes were positively or negatively correlated with the total wax coverage, amount of wax classes, or the relative abundance of homolog within each wax class (Figure 3). For example, positive correlation was observed between the relative expression level of *MrKCS1* and *MrCER1* and the total wax coverage and amount of primary alcohols. The relative expression level of *MrKCS1* was positively correlated with the abundance of C_28_ and C_30_ alcohol and C_30_ aldehyde, but was negatively correlated with C_26_ aldehyde. The relative expression level of *MrFAR3-2* was positively correlated with the relative abundance of C_29_ alkane, C_30_, and C_32_ primary alcohol.

### Diversities of the Coding Regions of the Wax Genes

To investigate the molecular mechanisms contributing to the wax variations among *M. ruthenica* populations, four genes involved in wax biosynthesis were obtained from cultivar cv TMT by RACE, including *MrFAR3-1*, *MrFAR3-2*, *MrCER1*, and *MrKCS1*, which were involved in the biosynthesis of primary alcohols, alkanes, and wax precursors, respectively. Furthermore, the CDS of the four wax genes were cloned from 30 populations, including the *MrFAR3-1* CDS from 25 populations, the *MrCER1* CDS from 11 populations, and the *MrKCS1* CDS from 30 populations (Table 2). No CDS could be cloned for *MrFAR3-2* except for TMT. From the cloned populations, *MrKCS1* had higher gene mutations, with polymorphic sites reaching 547, followed by *MrCER1* (61) and *MrFAR3-1* (22). Both Tajima’s test and Fu and Li’s test indicated that the values of the three wax genes were all lower than 0, with absolute value larger than 1.834 for Tajima’s *D* value, larger than 2.246 for Fu and Li’s *D* value, and larger than 2.442 for Fu and Li’s *F* value. Neutral evolution model test proved that the three wax genes did not fit the neutral evolution model, suggesting that the CDS of the three wax genes were naturally selected.

**TABLE 2:** Genetic polymorphisms of the coding sequences and promoter sequences of the wax biosynthesis genes from 30 *Medicago ruthenica* populations.

	Coding sequences			Promoter sequences			
	*MrKCS1* (30)	*MrCER1* (11)	*MrFAR3-1* (25)	*MrKCS1*	*MrCER1*	*MrFAR3-1*	*MrFAR3-2*
Sequence similarity (%)	91.12	99.65	99.88	97.73	96.53	94.80	93.85
Number of haplotypes	23	8	20	23	24	28	22
Haplotype diversity	0.97	0.927	0.949	0.982	0.984	0.995	0.966
Nucleotide diversity	0.029	0.009	0.002	0.005	0.011	0.011	0.005
Number of polymorphic sites	547	69	22	48	68	75	46
Number of singleton variable sites	527	61	16	19	39	34	25
Number of parsimony informative sites	20	8	6	29	29	41	21
Tajima’s *D* value	−2.803	−1.873	−1.834	−1.164	−1.02	−1.243	−1.567
Fu and Li’s D* value	−5.344	−2.246	−2.787	−0.939	−1.02	−0.939	−2.39
Fu and Li’s F* value	−5.314	−2.442	−2.919	−1.196	−0.713	−1.196	−2.502
Neutral evolution model test	*P* < 0.05	*P* < 0.05	*P* < 0.05	*P* > 0.10	*P* > 0.10	*P* > 0.10	*P* > 0.10

*The number in parentheses after the gene name was the number of populations successfully amplified. No coding sequence was amplified for *MrFAR3-2* except TMT.*

To test how the CDS mutations influence the gene functions, we analyzed the SNP sites using SnapGene; then, the amino acids and their isoelectric point, polarity, and hydropathy index were further analyzed. The results indicated that parts of the mutations were synonymous mutations, whereas certain numbers of mutations were missense mutations, resulting in the alterations of amino acids (Table 3 and Supplementary Excel 1). Though most of the amino acids from missense mutations had similar functional characteristics with the original amino acids, the isoelectric points, polarities, and hydropathy index of some amino acids were altered. Among the populations, overall, more SNPs were observed in the three wax genes of TMT when compared with the other populations.

**TABLE 3:** Selected missense mutations observed in coding sequences of wax genes in *Medicago ruthenica* (detailed mutations could be obtained in Supplementary Excel 1).

Population	Gene name	SNP position (bp)	Characteristics of amino acid in mutated population						Characteristics of amino acid in most population					
			SNP	Codon	Amino acid	Isoelectric point	Hydropathy index	Polarities	SNP	Codon	Amino acid	Isoelectric point	Hydropathy index	Polarities
WT003	*MrKCS1*	−92	G	CGA	Arginine	10.76	−4.5	Alkaline	A	CAA	Glutamine	5.65	−3.5	Polar neutral
02929	*MrKCS1*	−280	T	TTC	Phenylalanine	5.48	2.8	Nonpolar	G	GTC	Valine	5.96	4.2	Nonpolar
02254	*MrKCS1*	−280	C	CTC	Leucine	5.98	3.8	Nonpolar	G	GTC	Valine	5.96	4.2	Nonpolar
02930	*MrKCS1*	−280	C	CTC	Leucine	5.98	3.8	Nonpolar	G	GTC	Valine	5.96	4.2	Nonpolar
02914	*MrKCS1*	−280	A	ATC	Isoleucine	6.02	4.5	Nonpolar	G	GTC	Valine	5.96	4.2	Nonpolar
02906	*MrKCS1*	−283	G	GAA	Glutamic acid	3.22	−3.5	Acidic	C	CAA	Glutamine	5.65	−3.5	Polar neutral
02930	*MrKCS1*	−1531	T	TTC	Phenylalanine	5.48	2.8	Nonpolar	G	GTC	Valine	5.96	4.2	Nonpolar
WT003	*MrCER1*	−187	C	CTT	Leucine	5.98	3.8	Nonpolar	G	GTT	Valine	5.96	4.2	Nonpolar
2923	*MrCER1*	−187	C	CTT	Leucine	5.98	3.8	Nonpolar	G	GTT	Valine	5.96	4.2	Nonpolar
B5448	*MrCER1*	−363	G	ATG	Methionine	5.74	1.9	Polar neutral	A	ATA	Isoleucine	6.02	4.5	Nonpolar
2900	*MrCER1*	−363	G	ATG	Methionine	5.74	1.9	Polar neutral	A	ATA	Isoleucine	6.02	4.5	Nonpolar
WT003	*MrCER1*	−988	T	TTG	Leucine	5.98	3.8	Nonpolar	A	ATG	Methionine	5.74	1.9	Polar neutral
02930	*MrCER1*	−1369	C	CTT	Leucine	5.98	3.8	Nonpolar	A	ATT	Isoleucine	6.02	4.5	Nonpolar
02940	*MrCER1*	−1827	C	CAC	Histidine	7.59	−3.2	Polar neutral	A	CAA	Glutamine	5.65	−3.5	Nonpolar
00406	*MrFAR3-1*	−100	C	CTA	Leucine	5.98	3.8	Nonpolar	A	ATA	Isoleucine	6.02	4.5	Nonpolar
02913	*MrFAR3-1*	−107	C	GCT	Alanine	6	1.8	Nonpolar	T	GTT	Valine	5.96	4.2	Nonpolar
B5449	*MrFAR3-1*	−264	T	TTT	Phenylalanine	5.48	2.8	Nonpolar	G	TTG	Leucine	5.98	3.8	Nonpolar
02913	*MrFAR3-1*	−1228	A	AGT	Serine	5.68	−0.8	Polar neutral	C	CGT	Arginine	10.76	−4.5	Alkaline
02910	*MrFAR3-1*	−1259	A	AAC	Asparagine	5.41	−3.5	Polar neutral	G	AGC	Serine	5.68	−0.8	Polar neutral
02910	*MrFAR3-1*	−1360	G	GCA	Alanine	6	1.8	Nonpolar	T	TCA	Serine	5.68	−0.8	Polar neutral
02910	*MrFAR3-1*	−1378	A	ATA	Isoleucine	6.02	4.5	Nonpolar	G	GTA	Valine	5.96	4.2	Nonpolar

### Promoter Diversities of the Four Wax Genes

To investigate whether the wax variations among *M. ruthenica* populations were also regulated by mutations and methylation of promoter sequence, the promoters of *MrFAR3-1*, *MrFAR3-2*, *MrCER1*, and *MrKCS1* were obtained by using the chromosome walking method. Using DnaSP, we analyzed the haplotype diversity index, the nucleotide polymorphism index, and the neutral evolutionary algorithm model of the four promoters from 30 populations (Table 2). The number of polymorphic sites ranged from 46 to 75, and the nucleotide diversity ranged from 0.005 to 0.113, suggesting that the promoter regions of the four tested wax genes were genetically modified. Tajima’s *D* test indicated that the values were all lower than 0, with absolute value ranging from 1.164 to 1.567. Fu and Li’s test also indicated that the *D* and *F* values were lower than 0. The results indicated that the four promoters evolved under a neutral evolution model, suggesting that these promoters were not naturally selected.

Next, we predicted the acting elements of the four promoters using PlantCare (see text footnote 1). The main acting elements included core promoter element such as CAAT-box and TATA-box; light response elements such as GATA-motif, AT1-motif, and Gap-box; stress response elements such as LTR, MBS, and ARE; hormone-inducible elements such as CGTCA-motif/TGACG-motif, TCA-element, ABRE, and TGA-box; and other elements such as circadian, MSA-like, Myb, MYC, and AAGAA-motif (Supplementary Table 5). Compared among *M. ruthenica* populations, not much difference could be observed in the numbers and types of acting elements of the four promoters.

To test the possibility of promoter methylation in regulating gene expression, CpG islands were predicted using MethPrimer-PrimerDesign. Under the criteria of CG percentage > 50% and O/E > 0.6, no CpG island could be observed in *MrFAR3-1*, *MrFAR3-2*, and *MrCER1* from all populations, except for a 99-bp CpG at the initial region of *MrKCS1* (data not shown), which was lower than the minimum requirement of 300 bp for normal CpG island. Furthermore, DNA methylation sites on promoter region were proved by bisulfite sequencing, and no DNA methylation site could be observed on the promoters of the four wax genes (data not shown).

### Responses of Leaf Cuticular Waxes to Simulated Environmental Conditions

**FIGURE 4:**
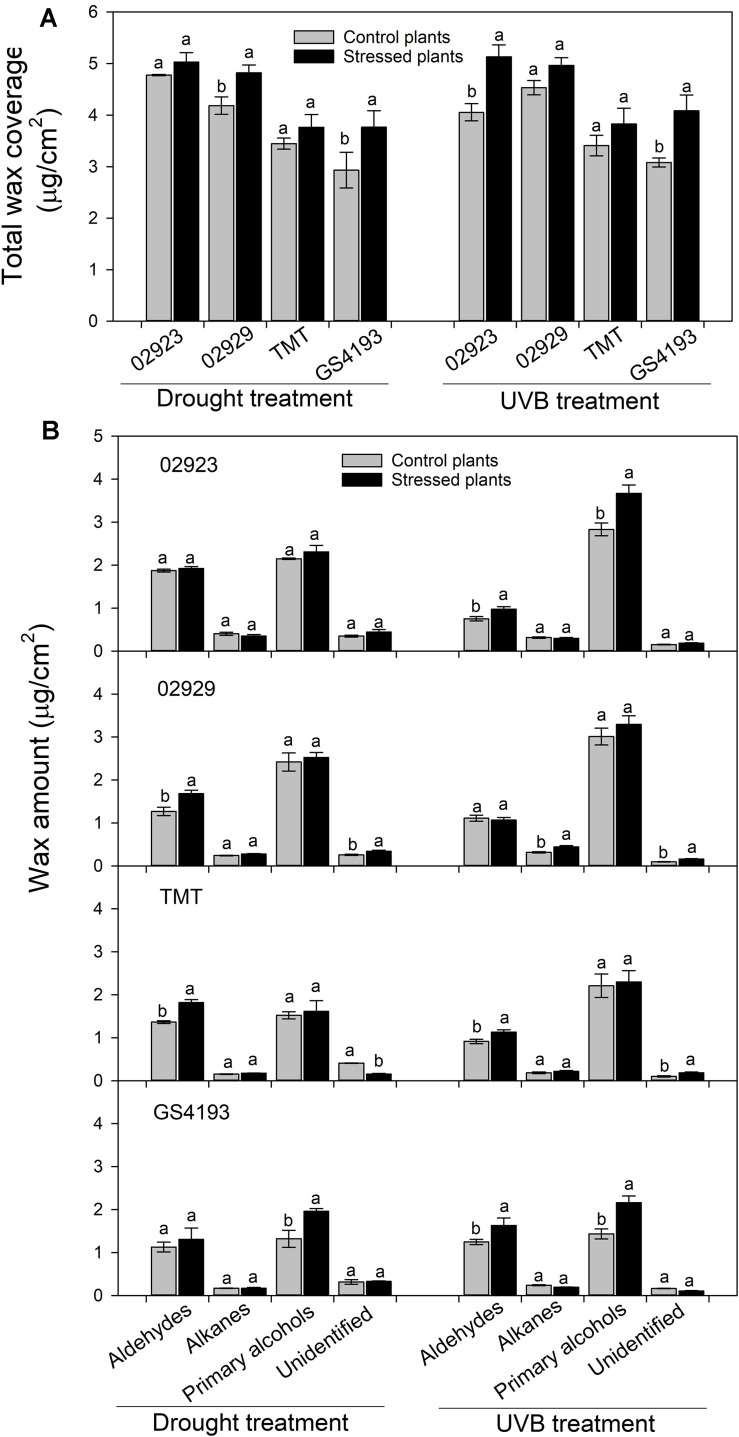
The wax coverage **(A)** and relative abundance of wax compositions **(B)** of *Medicago ruthenica* under enhanced UVB and drought treatments. Different lowercase letters above the data bar within each wax class represented significance at *P* < 0.05 according to Least Significant Difference test.

**FIGURE 5:**
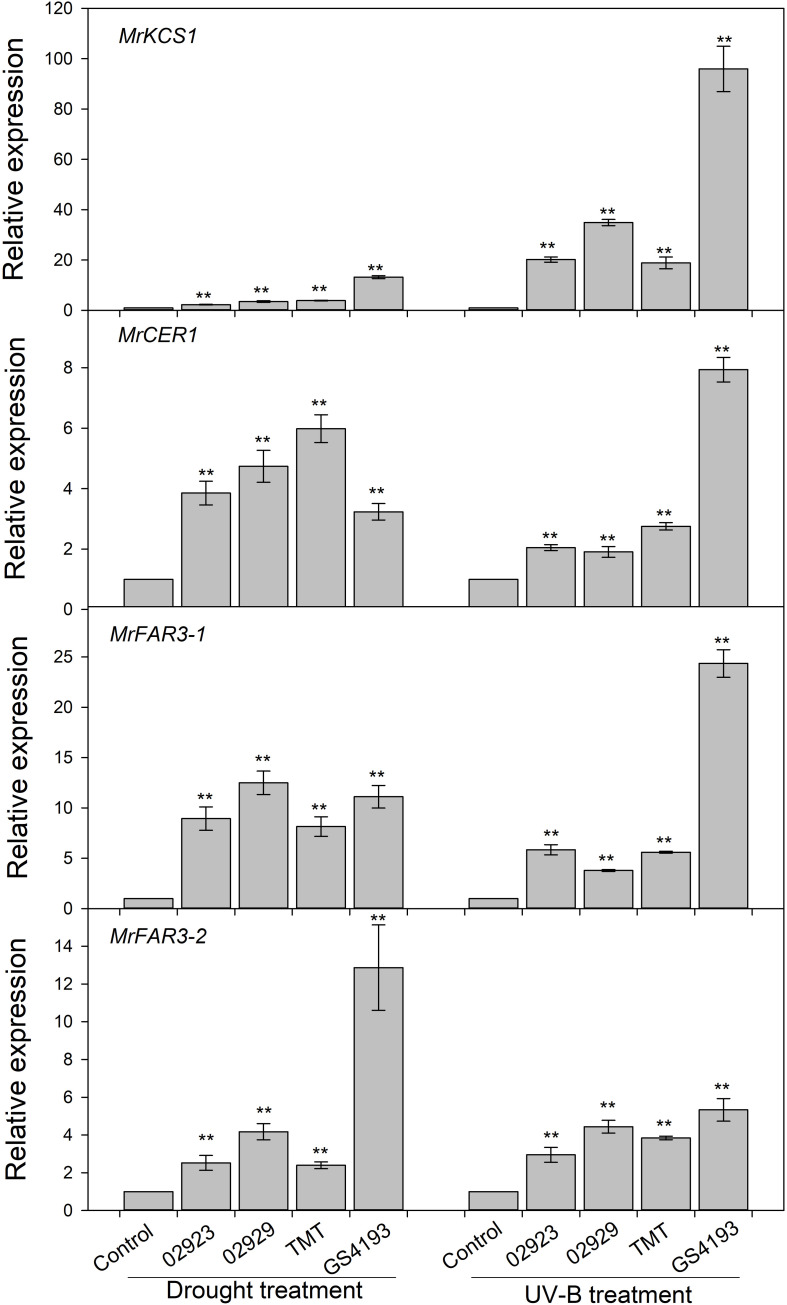
Relative expressions of four wax genes under drought and enhanced UV-B treatments. ** represented significance when compared with the control (the control for each cultivar was adjusted to one) using Student’s *t* test.

To test the phenotypic plasticity responses of leaf cuticular wax under varied environmental conditions, two populations with relatively higher wax amounts (02923 and 02929) and two populations with relatively lower wax amounts (TMT and GGS4193) in the common garden pot experiment were further treated with enhanced UVB and water deficit. The total wax coverage in drought-stressed plants was higher than those of the well-watered plants, except for insignificant changes in 02923 and TMT under drought treatment and in 02929 and TMT under UVB treatment (Figure 4). Drought treatment significantly increased the amounts of aldehydes in 02929 and TMT and the amounts of primary alcohols in GS4193, whereas UVB treatment significantly increased the amounts of aldehydes and primary alcohols in 02923 and GS4193. An increase of alkanes was observed in UVB-treated 02929. qRT-PCR analysis indicated that the expression levels of the four wax genes increased under both drought and UVB treatments (Figure 5). Overall, no obvious difference in their responses of wax amounts and expression levels to drought and UVB treatments could be observed between two high-wax and two low-wax plant populations.

## Discussion

### Variations of Cuticular Waxes Among Populations Were Induced by Their Original Environments

During the evolution process, the plants exhibit certain phenotypes under similar environmental conditions (Henn et al., 2018). In this study, seeds of 30 populations of *M. ruthenica* were sampled from their field-growing environments. These populations showed great variations of seed and leaf morphological properties (Supplementary Table 2), suggesting that the growing conditions changed their phenotypes. Cuticular wax has also been shown to be related to plant’s adaptations to changing environments (Yeats and Rose, 2013). In the common garden pot experiment, under the same growing conditions, the leaf cuticular waxes showed great variations among populations. For example, total wax coverage ranged from 1.06 to 6.11 μg/cm^2^, with CV reaching 36.57%. Similar CV was also observed for the amounts of alkanes, primary alcohols, and aldehydes. A study on 59 populations of *L. chinensis* in a common garden experiment has also shown that great intraspecific trait variations were observed for total wax coverage and wax compositions (Li et al., 2020). Andrae et al. (2019) also reported that *M. quinquenervia* had evolved their specific wax characteristics to adapt to their surroundings. These results suggest that plant can adjust its wax depositions when subjected to different environmental pressures. Such intraspecific trait variations among plant populations growing under different environmental conditions provided implications for understanding global change responses (Moran et al., 2016).

Drought and enhanced UVB stresses increased the total wax coverage as well as the relative expression levels of the four wax genes in both two high-wax and two low-wax populations of *M. ruthenica*. This further validated that *M. ruthenica* would alter the leaf wax deposition when facing climate changes, which contributed to their improved stress adaptations (Shepherd et al., 1997; Gordon et al., 1998; Kosma et al., 2009). During the last two decades, great achievements have been obtained in understanding the responses to leaf cuticular wax to environmental stresses like drought (Kosma et al., 2009), cold (He et al., 2019), light (Giese, 1975), enhanced UVB (Pilon et al., 1999), and high temperature (Shepherd et al., 1997). To test how much the environment factors contribute to such variations of cuticular waxes among *M. ruthenica* populations, Pearson’ correlation analysis and redundancy analysis were applied. However, no significant correlation between climate factors and the wax characteristics could be observed (data not shown). This was inconsistent with the results from *L. chinensis* where latitude, arid index, and the precipitation had been shown to be the most important climate parameters contributing to the variations of the total wax coverage and the amount of wax compositions (Li et al., 2020). In a common garden experiment of *L. chinensis*, rhizomes were collected from their field-growing environments and transplanted. In this study, seeds were collected and used. Besides the difference in plant species, asexual generations might keep more heretic information related to growing conditions from their host plants, whereas some phenotypes formed during growing conditions might be lost through sexual reproduction (Bengtsson, 2003). No matter what, great variations of cuticular waxes in the common garden experiments did imply that the wax deposition characteristics formed in their original growing conditions could be inherited.

### Genetic Basis of the Variations of Leaf Cuticular Waxes Among Populations

Pearson’s correlation analysis indicated that the expression levels of the four wax genes were positively or negatively correlated with the total wax coverage, amounts of wax classes, or the relative abundance of homolog within each wax class, suggesting that the variations of leaf cuticular wax were related to the gene transcriptional level. There were two possibilities contributing to the variations of the leaf cuticular waxes among populations of *M. ruhenica* in the common garden experiment, genetic variations and epigenetic modifications. As the genes involved in primary alcohol biosynthesis in *M. ruthenica*, *MrFAR3-1* and *MrFAR3-2* had 74% similarity for CDS. However, using DNA from TMT as the template, no CDS could be amplified for *MrFAR3-2* from other populations, implying that the CDS might be mutated greatly, particularly the sequences of upstream and downstream. In total, 22 polymorphic sites were observed for *MrFAR3-1*, 69 for *MrCER1*, and 547 for *MrKCS1*. Both Tajima’s test and Fu and Li’s test further indicated that the CDS of the three wax genes were not matching the neutral evolution model (Ohta, 2003), suggesting that these wax genes were naturally selected (Kreitman and Akashi, 1995). A study on *Cryptomeria japonica* has shown that 43 out of 208 outlier loci were associated with environmental variables (Tsumura et al., 2014). Lu et al. (2019) also reported that environment-associated SNPs composed the genetic structure of adaptive phenotypic traits including height, diameter, metabolite levels, and gene transcript abundance of *Pinus taeda* L. These results further suggested that long-term growth under certain environments would induce mutations in coding regions of the wax genes, which might influence the function/activity of the enzymes encoded by these genes (Zeng et al., 2017), and thus modulate the wax biosynthesis and depositions. Analysis of the mutated regions on nucleotide sequence further proved that some of the missense mutations altered the amino acid as well as their isoelectric points, polarities, and hydropathy index. A study with two lignin biosynthesis genes in *E. globulus* has also shown that polymorphism affecting highly conserved amino acids may alter enzyme function, and this molecular variation may be linked to the variation in lignin profiles (Poke et al., 2003). Further studies are needed to clarify how the mutated positions influence the gene functions and the contributions of each mutated position in influencing the gene function. Therefore, the genetic mutations of the coding region might be driven by the environment changes, fitting with the adaptive evolution of *M. ruthenica*, and the induced alterations of the phenotypes of cuticular wax contributed to the improvements of plant adaptations to their original growing environments (Dodd and Poveda, 2003; Shepherd and Griffiths, 2006).

A number of polymorphic sites were also observed on the tested promoters, suggesting that the promoter regions of these wax genes were genetically modified. However, both Tajima’s test and Fu and Li’s test indicated that the promoters of the four wax genes showed neutral evolution and the most modified nucleotides were the low-frequency alleles, suggesting that these promoters were not naturally selected (Simonsen et al., 1995). Meanwhile, not much difference among 30 populations could be observed in the numbers and types of the acting elements that related to the gene expression such as light response elements, hormone-inducible elements, stress response elements, and other elements like MYB and MYC (Kuhlemeier, 1992; Yamaguchi-Shinozaki and Shinozaki, 2005). This implied that the genetic variations of the promoter region might not be related to the variations of the gene expression of the four wax genes among *M. ruthenica* populations.

In recent years, studies have shown that epigenetic variations can help natural populations cope with their growing environments (Thiebaut et al., 2019), and inheritable epigenetic mutations (epimutations) can contribute to transmittable phenotypic variations (Wang and Fan, 2015). In this study, the percentage of CpG islands and O/E ratio did not match the requirements for DNA methylation (Gardiner-Garden and Frommer, 1987), suggesting that the promoters of the wax biosynthesis genes were not methylated. This implied that DNA methylation of the promoter sequences might not be involved in regulating the wax gene expressions, and thus the cuticular wax biosynthesis. To clarify the contributions of epigenetic modifications in regulating wax gene expressions, other epigenetic modifications need to be further analyzed such as histone modification, chromatin remodeling, and non-coding RNA. Detailed exploration of the contributions of epigenetic modifications in regulating wax gene expressions might lead to an appreciation of how new phenotypes could be generated quickly in response to environmental modifications (Chang et al., 2020).

Altogether, we provide evidence that both environments and genetic variations contribute to the variations of the leaf cuticular waxes among populations of *M. ruthenica* originated from different growing environments. We found that the total wax coverage and the abundance of wax classes and wax compounds varied greatly among populations in common garden experiment, which was further proved to be the adaptive responses of the leaf cuticular waxes to growing environments in two stress-modeling experiments. Cuticular waxes are sensitive to changing environments and have been shown to be related to plant adaptations. The analysis of SNP in CDS indicated that some of the missense mutations altered the amino acid as well as their phy-chemical properties, suggesting that genetic mutations might have altered the phenotypes of cuticular wax. The numbers and types of acting elements in promoter sequences were similar among the tested populations, and no DNA methylation could be observed in the promoter sequences, suggesting that variations of the polymorphic sites in promoter sequences and DNA methylation were not involved in regulating wax gene expressions. Tajima’s test and Fu and Li’s test indicated that the promoters of the wax genes showed neutral evolution model and were not naturally selected, whereas CDS of the wax genes were not matching the neutral evolution model and were naturally selected. Therefore, we conclude that growing environments will induce mutations of the coding region of the wax biosynthesis genes, which contributes to the variations of the gene expressions, and thus the cuticular wax biosynthesis and depositions.

## Data Availability Statement

The datasets presented in this study can be found in online repositories. The names of the repository/repositories and accession number(s) can be found in the article/ Supplementary Material.

## Author Contributions

YN and YG contributed to study conception and design. YL, XiZ, and YN collected data. YW, XuZ, ZL, and QX analyzed the data. YG wrote the manuscript with contributions from all authors.

## Conflict of Interest

The authors declare that the research was conducted in the absence of any commercial or financial relationships that could be construed as a potential conflict of interest.

## Footnotes

1
http://bioinformatics.psb.ugent.be/webtools/plantcare/html/

2 http://www.urogene.org/methprimer/

3 https://www.snapgene.com

## Supplementary Material

Supplementary Table 1 Environmental factors where seeds of each population of *Medicago ruthenica* were collected.

Supplementary Table 2 Variance analysis of leaf and seed morphological indexes of *Medicago ruthenica* populations.

Supplementary Table 3 Correlation analysis of original environmental factors with seed and leaf characters.

Supplementary Table 4 Primers used in this study.

Supplementary Table 5 *Cis*-acting element of the promoter sequence of *MrFAR3-1*, *MrFAR3-1*, *MrCER1*, and *MrKCS1*.
