# Supplementary material for: Environment-Driven Adaptations of Leaf Cuticular Waxes Are Inheritable for Medicago ruthenica
Source: Front Plant Sci. 2021 May 17;12:620245. doi: 10.3389/fpls.2021.620245 (PMC8165318; doi:10.3389/fpls.2021.620245)
Supplement: Supplementary Table 1 — Environmental factors where seeds of each population of Medicago ruthenica were collected. [file Data_Sheet_4.pdf]

Table S1 Environmental factors where seeds of each population of *Medicago ruthenica* were collected.

No	Population	Location	Longitude	Latitude	Soil	AMT(°C)	AMP(mm)
1	GS3734	Long county, Shannxi	106.87	34.898	Yellow cinnamon soils	10.7	600.1
2	GS4193	Huangling, Shannxi	109.36	35.623	Yellow cinnamon soils	9.4	568.8
3	2914	Huhhot, Inner Mongolia	111.027	40.839	Brown pedocals	6.7	400
4	2926	Tumote Zuoqi, Inner Mongolia	111.223	40.738	Meadow soils	3.3	400
5	2925	Tumote Zuoqi, Inner Mongolia	111.299	40.744	Meadow soils	3.3	400
6	912	Qingshuihe, Inner Mongolia	111.643	39.926	Castano-cinnamon soils	7.1	400
7	2924	Damao Qi, Inner Mongolia	111.277	41.336	Brown pedocals	3.6	250
8	2923	Wuchuan, Inner Mongolia	111.213	40.909	Meadow soils	2	450
9	2900	Mongolia	111.758	40.849	Brown pedocals	6.7	400
10	406	Huhhot , Inner Mongolia	111.68	40.805	Castano-cinnamon soils	2.5	400
11	2940	Huitengliang, Inner Mongolia	112.956	41.149	Sand soil	4	360
12	2254	Datong county, Shanxi	113.491	40.062	Sand soil	5.8	392
13	TMT	Tumote Zuoqi, Inner Mongolia	112.006	43.635	Sand soil	6.3	400
14	2911	Wulanchabu, Inner Mongolia	113.134	41.042	Brown pedocals	5.7	980
15	2912	Siziwang Qi, Inner Mongolia	113.115	41.038	Sand soil	6	350
16	B5449	Dehua county, Inner Mongolia	114.016	41.91	Sand soil	1.9	334
17	Z-2157	Yanqing county, Beijing	115.906	40.534	Sand soil	8	477.2
18	15616	Subsurb, Beijing	116.39	39.905	Cinnamon soils	11.3	750
19	532	Yang county, Liaoning	118.883	38.716	Sand soil	10.4	500
20	WT-003	Xilinghot, Inner Mongolia	116.032	43.924	Castanozems	4	360
21	B5448	Zhenglan Qi, Inner Mongolia	116.035	42.258	Castanozems	1.5	365
22	B5447	Weichang County, Hebei	117.765	41.944	Sand soil	13	454
23	2901	Xilingol, Inner Mongolia	116.054	43.939	Castanozems	4	360
24	2907	Baiyinxile, Inner Mongolia	116.641	43.717	Meadow soils	-0.2	345
25	2910	Baiyinxile, Inner Mongolia	116.643	43.722	Meadow soils	-0.2	345
26	2930	Balin Youqi, Inner Mongolia	118.674	43.542	Castanozems	4.9	359
27	2913	Baiyinxile, Inner Mongolia	118.68	43.536	Meadow soils	-0.2	345
28	2251	Manzhouli, Inner Mongolia	117.364	49.575	Dark chestnut soil	0.7	300

29	2936	Naiman Qi, Inner Mongolia	120.898	44.562	Meadow soils	6.4	360
30	2929	Naiman Qi, Inner Mongolia	120.89861	44.562	Meadow soils	6.4	360

AMT, annual mean temperature; AMP, annual mean precipitation.

Table S2 Variance analysis of leaf and seed morphological indexes of *Medicago ruthenica* populations

	Min.	Max.	Ave.	CV%
Seed length（mm）	2.06	3.04	2.47	7.69
Seed width（mm）	1.63	2.05	1.84	5.23
Seed length/ width	1.06	1.62	1.35	7.07
Thousand seed weight（g）	2.08	3.34	2.60	10.91
Leaf length（cm）	0.65	1.48	1.07	15.72
Leaf width（cm）	0.40	1.11	0.72	20.21
Leaf length/ width	1.12	3.40	1.55	24.22
Leaf area（cm^2）	1.23	3.76	2.02	25.64
Leaf angle（°）	30.06	77.75	60.08	17.46

Table S3 Correlation analysis of original environmental factors with seed and leaf characters

Parameters	Longitude	Latitude	Annual mean temperature	Annual mean precipitation
Leaf length	0.536**	-0.540**	-0.040	-0.168
Leaf width	-0.197	-0.234	0.278	0.134
Leaf length/ width	-0.276	-0.253	-0.214	-0.222
Leaf area	-0.516**	-0.514**	0.040	-0.205
Leaf angle	0.022	0.025	0.086	0.089
Seed length	-0.075	-0.013	-0.325	-0.328
Seed width	-0.047	-0.052	0.032	-0.147
Seed length/ width	-0.051	0.023	-0.388*	-0.261
Thousand seed weight	-0.130	-0.110	-0.186	-0.271

*, P<0.05; **, P<0.01.

Table S4 Primers used in this study

Genes	Primer sequence（5′ to 3′）
Rapid amplification of cDNA ends (RACE) for coding sequence from TMT	
RACE Kit Primers	5' RACE Abridged Anchor Primer:
	GGCCACGCGTCGACTAGTACGGGGGGGGGGGGG
	5' RACE AUAP: GGCCACGCGTCGACTAGTAC
	3' RACE Universal Primer Mix:
	CTAATACGACTCACTATAGGGCAAGCAGTGGTATCAACGCAGAGT
	CTAATACGACTCACTATAGGGC
	3' RACE Nested Universal Primer: AAGCAGTGGTATCAACGCAGAGT
MrCER1	5' RACE GSP1: TGGATGTATCACAGAAGT
	5' RACE GSP2: AACAATGGAAGAATGATGATG
	5' RACE GSP3: TAGAATAAAGGAAATGATG
	3' RACE GSP1: CACACCAGCCATGATAACTCCCCCTA
	3' RACE GSP2: TATGAGTGCATGGCGTATTGCTGGAA
MrKCS1	5' RACE GSP1: GCTTGTATTTGGACCG
	5' RACE GSP2: TTGGAAAGAAGTATAGCGGC
	5' RACE GSP3: GAACGATCATTTCCAAAGTAC
	3' RACE GSP1: TGGAACCGTCTCGGATGACTCTGC
	3' RACE GSP2: GTATTCATGCTGGTGGAAGAGCTGTT
MrFAR3-1	5' RACE GSP1: TTTCTGAGATAAAGCT
	5' RACE GSP2: CATGATTTTCCTTCAGCA
	5' RACE GSP3: ATCTCATTGTGCATTCTTCT
	3' RACE GSP1: ACCTACAATCATTACCAGCACTTA
	3' RACE GSP2: AGTAAGAACCATAGACAGTTTAATTG
MrFAR3-2	5' RACE GSP1: TTTCCGAAAGAAATGG
	5' RACE GSP2: CCTAATTTTTCCTTTAGAC
	5' RACE GSP3: TCTCATTGTGAAAGCGATG
	3' RACE GSP1: TCCAGCCATTGTCACTAGTACATT
	3' RACE GSP2: TGTAAGGACTATTGATAGTTTAGCC
Chromosome Walking for promoter sequences from TMT	
MrCER1	SP1: GCTATGAATGGTGTCAAAATCACC
	SP2: GATAAATAATTGGAATTATGC
MrKCS1	SP1: GAATGGTTGTGGCGTTGCATGAAT(SP1)
	SP2: CATAACCTAACTTTACGTAC(SP2)
MrFAR3-1	SP1: AAGTAAACACATATTAATTTGAAT(SP1)
	SP2: TATGCACACAAGTAAGTA(SP2)
MrFAR3-2	SP1: AAGAAAAGAAAAACATAAAAAGG(SP1)
	SP2: GCCAGTGGCACCAGTGAC(SP2)
Primers used to clone promoter sequences	
MrCER1	F: TCAAACCAGGTAAAGATGAAGTAGC
	R: GGCCAATCACTAAGTATGCCAG
MrKCS1	F: GACAAGTTATGAGGCGACGATG
	R: GTAAGAGAGGGACGACGAGTGTG
MrFAR3-1	F: ACAGAAGGCAATCCAGACTCG

	R: TTACTCTTTGCAAGGAAGCCAG
MrFAR3-2	F: TTTAAGCTTTTCTGCTATGGTGG
	R: AGTGGCACCAGTGACTAAAACG
Primers used to clone coding sequence	
MrCER1	F: ATGGCGTCCAAACCTGGCATA
	R: TCAATCAAGGGGATGATTAATCTTCAA
MrKCS1	F: ATGAGTCGCAACAGCATAGACATGG
	R: TTAAGAAGCAACAGAAACAGGGACACTA
MrFAR3-1	F: ATGGATCAATTTGGAAGTATTGCTCAC
	R: TCACTTGAAGGAATATTTGACAATGCC
MrFAR3-2	F: ATGGAGTTGGGAAGTGTACTCCATTT
	R: TCACTTTAGAATGTATTTAACGATGCCAG
Primers used for fluorescent quantitative PCR	
Actin	QF: CCACATGCCATCCTTCGTTT
	QR: TGTCACGGACAATTTCCCG
MrCER1	QF: TTTAGGTCTTTCAAACCAGGGAG
	QR: CCAAGCTACTGCCATCAACAATC
MrKCS1	QF: CATTTTGTCAAGAGCAGGTTTAGG
	QR: AAAAGTGCGTCTAATGATCCAAAC
MrFAR3-1	QF: GTCCATACTGTGATGCGGTTG
	QR: AATGCCAGGATTATGGATGTTC
MrFAR3-2	QF: CGGTTGGTAGACCTCTACAAGCC
	QR: ACAAATCTGTCTCCACCCCACC

Table S5 Cis-acting element of the promoter sequence of *MrFAR3-1*, *MrFAR3-1*, *MrCER1*, and *MrKCS1*

Regulatory elements	Sequence	Function	Quantity in promoter			
			MrKCS1	*MrCER1*	*MrFAR3-1*	*MrFAR3-2*
GT1-motif	GTGTGTGAA/GGTTAA	light responsive element	0	0/1	1	2
MrE	AACCTAA	MYB binding site involved in light responsiveness	1	0	1	0
G-box	CACGAC	involved in light responsiveness	2/3	0	1	0
3-AF1 binding site	TAAGAGAGGAA	light responsive element	0/1	0/1	0	0
LTR	CCGAAA	involved in low-temperature responsiveness	0	0/1	0/1	0
MBS	CAACTG	MYB binding site involved in drought-inducibility	2/3	1	0/1	0/1
ARE	AAACCA	essential for the anaerobic induction	4/5	1	0	2/3
CGTCA-motif	CGTCA	involved in the MeJA-responsiveness	1	0	2/3	0
TGACG-motif	TGACG	involved in the MeJA-responsiveness	1	0	2/3	0
TGA-element	AACGAC	auxin-responsive element	0	0	0	1
ABRE	TACGGTC	involved in the abscisic acid responsiveness	0	1	0	0
TCA-element	CCATCTTTTT	involved in salicylic acid responsiveness	2	0/1	0	0
circadian	CAAAGATATC/	involved in circadian control	0/1	0	1	0/1
MSA-like	TCAAACGGT	involved in cell cycle regulation	0	0	0/1	0
GCN4_motif	TGAGTCA	involved in endosperm expression	0	0	0/1/2	0
CAT-box	GCCACT	related to meristem expression	0	0	0	1
MBSI	TTTTTACGGTTA	MYB binding site involved in flavonoid biosynthetic genes regulation	0	1	0	0
O2-site	GATGATGTGG	involved in zein metabolism regulation	0/1	0	0	0

The value was the number of acting element observed from 30 populations.
